# Is Nutritional–Behavioral Intervention Effective in the Fight Against Obesity?

**DOI:** 10.3390/nu18172814

**Published:** 2026-08-27

**Authors:** Agnieszka Momora, Izabela Sałacińska, Andrea Sollárová, Paweł Więch

**Affiliations:** 1 Postgraduate Studies Centre, Medical College, University of Information Technology and Management, 35-225 Rzeszow, Poland; agnieszkamomora@gmail.com; 2 Institute of Nursing, Faculty of Health Sciences and Psychology, University of Rzeszow, 35-310 Rzeszow, Poland; pwiech@ur.edu.pl; 3 University Centre for Research and Development in Health Sciences, University of Rzeszow, 35-959 Rzeszow, Poland; 4 Department of Nursing, Faculty of Social Sciences and Health Care, Constantine the Philosopher University in Nitra, 949 01 Nitra, Slovakia; asollarova@ukf.sk

**Keywords:** weight reduction, diet therapy, remission of obesity, obesity-related diseases

## Abstract

Background/Objectives: Obesity is a complex systemic condition driven by interactions between biological mechanisms and environmental factors. This retrospective, uncontrolled, completer study aimed to evaluate changes in body composition and long-term weight maintenance, as well as the impact of comorbidities, in adult patients undergoing personalised dietary and behavioural interventions. Methods: A retrospective analysis of outpatient medical records (2018–2024) was conducted. Of the 1416 patients analysed, 267 overweight or obese adults completed the active treatment phase and a one-year post-intervention follow-up period. The primary endpoints were absolute changes in body weight, BMI, and body fat mass (BFM), as measured by bioelectrical impedance analysis (BIA), as well as the maintenance of ≥10% weight loss after one year. The intervention consisted of a personalised diet based on a food-exchange system, combined with psychodietetic support. Results: Participants achieved a mean weight loss of 10.45 ± 7.56 kg, which was associated with significant decreases in BMI and BFM. The average loss of fat-free mass and skeletal muscle mass was 1.72 ± 2.20 kg and 1.11 ± 1.36 kg, respectively. Weight maintenance, defined as maintaining a weight loss of at least 10% of baseline body weight after a one-year follow-up period, was achieved by 23.2% of participants who completed the study. Conclusions: Participants experienced significant reductions in body weight and body fat while preserving lean body mass to a moderate extent. However, long-term maintenance of weight loss was achieved by less than a quarter of participants after one year, highlighting the ongoing challenge of weight regain.

## 1. Introduction

The modern obesity epidemic is inextricably linked to ongoing globalization processes. Research indicates that an increase in the globalization index by one standard deviation correlates with nearly a 25% expansion in the obesity rate, making the phenomenon of ‘globesity’ one of the most serious civilizational challenges [1], with global health metrics confirming its growing impact on mortality and morbidity [2]. The dynamic evolution of contemporary therapeutic paradigms—spurred by the recognition that obesity requires placement within a much wider public health and clinical context [3]—is reflected in the latest expert consensus [4,5]. Despite intensive public health efforts aimed at combating the obesity epidemic, the effectiveness of traditional methods remains limited, as only 20% of patients achieve long-term disease remission [6]. This crisis also has a macroeconomic dimension. Projections suggest that by 2060, the cost dynamics related to overweight and obesity will reach an unprecedented level, especially in low-income countries, where their estimated increase is 12 to 25 times [7]. At the same time, Okunogbe and colleagues demonstrate that implementing effective countermeasures could reduce obesity prevalence by 5% annually, saving the global economy approximately 429 billion USD each year [7]. In response to these challenges, the current therapeutic approach to treating obesity incorporates lifestyle modifications and advanced intervention strategies. Dietary intervention, physical activity and psychodietetic support form the basis of conservative treatment, while pharmacotherapy and bariatric procedures are essential for intensified therapy in patients with severe obesity. Despite continuous advancements in surgical and pharmacological methods, a balanced dietary model remains essential for long-term metabolic control and maintaining treatment outcomes. With such a broad spectrum of therapeutic options available, this study highlights the importance of an integrated nutritional and behavioural strategy aimed at optimising body composition and minimising the risk of weight regain [8]. However, the lack of a clear, repeatable methodology that guarantees sustained weight reduction continues to drive the search for innovative solutions. This work presents the results of a study evaluating the nutritional status of adults subjected to an integrated dietary–behavioral strategy. The aim of the analysis was to verify the method’s effectiveness in optimizing body composition and minimizing the risk of weight fluctuations after the completion of the therapeutic process. Additionally, a hypothesis was formulated that systematic dietary–behavioral intervention leads to a statistically significant improvement in body composition, with the dynamics of these changes being conditioned by the clinical profile of the patient. The presented work is a pioneering analysis of methodology developed since 2018. It is worth emphasizing that the adopted approach anticipated formal standards, demonstrating full substantive consistency with them.

## 2. Materials and Methods

### 2.1. Ethics

The study was conducted in accordance with the highest ethical standards outlined in the Declaration of Helsinki and in Polish national regulations. The study received a positive opinion from the Bioethics Committee of the State University of Applied Sciences in Przemyśl, No. 7/2023, appointed under Ordinance No. PANS-SEK-021/36/22 of the Rector of the State University of Applied Sciences in Przemyśl. All patients gave informed and voluntary consent to participate, which was confirmed by their participation. The study participants were also informed of its purpose and had the opportunity to withdraw from the study at any stage without providing a reason. As part of the safety monitoring of the intervention, a systematic assessment of potential adverse events was conducted. No clinically significant adverse events or side effects attributable to the dietary–behavioral strategy were identified across the entire cohort.

Between February 2018 and December 2024, patients underwent a long-term nutritional and behavioural intervention as part of standard outpatient care. Once data collection had been completed, a research protocol was developed and used to conduct a retrospective analysis of the medical records of patients who met specific inclusion criteria. The study was conducted at the Medyk Medical Centre in Rzeszów. All patients included in the analysis were under the care of a single qualified dietitian, who constituted their entire clinical population during the specified time period. The intervention was completely safe and well tolerated; none of the participants needed to stop or change the therapy due to adverse reactions. All patient data were anonymized to ensure their privacy and compliance with ethical standards of scientific research.

### 2.2. Research Questions and Hypothesis

The main goal of this study was to evaluate the effectiveness of a dietary and behavioral strategy in the treatment of adult patients with excess body weight. The first research issue addressed the impact of the implemented strategy on changes in body mass and composition, with the hypothesis that the implementation of the model would result in a statistically significant reduction in body weight and optimization of its composition parameters. Another aspect of the analysis focused on determining the extent to which comorbid conditions influence the effectiveness of the intervention. It was assumed that metabolic disorders and hormonal disturbances would correlate with lower therapy effectiveness. The final issue addressed the scale of body weight fluctuation within the studied cohort. It was hypothesized that the therapy model would minimize the risk of weight fluctuation over the long term.

### 2.3. Subjects

Each study participant completed a proprietary research questionnaire based on recognized, standardized, and validated psychometric tools: the Health Behavior Inventory (IZZ) developed by Z. Juczyński and the Generalized Self-Efficacy Scale (GSES). The use of these tools was crucial to the study’s objective. The IZZ Inventory enabled the assessment of an overall index of health-promoting behaviors (including dietary habits and preventive behaviors), while the GSES scale allowed for the estimation of the participants’ level of self-efficacy. The author’s strategy for working with patients was based on constructing individually tailored meal plans that were precisely adapted to the patients’ specific metabolic needs, dietary preferences, and health goals. The intervention was led by a qualified dietitian holding a master’s degree in dietetics (Collegium Medicum of the University of Rzeszów) and who had completed postgraduate studies in psychodietetics (WSB Merito University in Gdańsk). Recommendations regarding physical activity were also discussed with physiotherapists holding bachelor’s or master’s degrees. The proposed nutritional intervention model was developed based on guidelines contained in the Standards of Dietary Treatment for Simple Obesity in Adults from 2015 [9]. Each participant received a personalized dietary scheme built on food exchange lists, which allowed flexibility in meal composition and adaptation to individual menus. Thanks to this method, patients were not restricted to strictly following specific culinary recipes but had the opportunity to create their own dishes by choosing products consistent with their preferences. Initially, patients were required to weigh carbohydrate-containing foods such as bread, grains, rice, pasta, and potatoes, as well as protein products like dairy, meat, fish, or legumes. However, weighing fats such as olive oil or other oils was not necessary, as these products were measured using standard kitchen measures like tablespoons or teaspoons. This scheme also eliminated the need to weigh fruits and vegetables. It is worth noting that after implementing the strategy, most dishes were prepared based on standard kitchen measures. This was possible because patients memorized approximate weights of dry products (e.g., grains, rice, pasta, legumes) and liquids (e.g., milk, fermented dairy products, and their plant-based substitutes) after pouring or transferring them into a glass. During each dietary consultation, if needed, the meal plan was adjusted based on the patient’s subjective feelings of satiety or hunger, which were quantified using a hunger scale from 1 to 10. These modifications included the diet’s caloric content and the size of individual portions. Additionally, the meal plan was adjusted in response to observed metabolic adaptations. To overcome these adaptations, the dietitian implemented a strategy called a diet break. This break, lasting 2 to 4 weeks, involved temporarily adjusting the energy balance to a baseline energy requirement while maintaining the intended nutritional composition of the meals. If necessary, the patient also received new culinary recipes that could be integrated into the established meal plan. As part of the method, patients maintained constant communication with the dietitian, reporting their food intake daily and physical activity. Reporting was done through the submission of food and activity diaries, which could include photographs of meals, descriptions, and data from mobile apps or activity monitoring devices. Communication between patients and dietitians occurred via various channels such as SMS, online messaging apps, and email, ensuring continuous support and quick interaction.

Additionally, patients received psychodietetic tasks aimed at supporting the process of changing eating habits, which included reading professional literature, listening to podcasts, watching educational materials, or practicing affirmations. Patients also maintained a so-called “habit chain,” a tool to help build and reinforce desirable eating behaviors. A habit chain has been defined as a structured self-monitoring protocol designed to automate health-promoting behaviors. Typically, this tool involved monitoring the number of steps, fluid intake (including water), type and duration of physical activity, and consumption of foods outside the diet along with their quantities.

The psychodietetic studies were designed based on recognized psychotherapeutic approaches, including cognitive–behavioral therapy (CBT), acceptance and commitment therapy (ACT), and the task-focused approach (TSR). They included techniques for managing cravings and emotional hunger based on cognitive defusion (labeling impulses and the wave metaphor), cognitive restructuring with elements of self-compassion, and stimulus substitution and attention redirection. The concept of the Minimum, Optimum, and Maximum Plan was also implemented, eliminating “all-or-nothing” dichotomous thinking by flexibly adapting actions to the patient’s current psychophysical resources. Validated psychometric and health coaching tools, such as the Wheel of Life and Priority Analysis, were also used to evaluate life balance, strengthen self-efficacy, and identify personal resources.

The process of tailoring the strategy was based on a structured interview and ongoing evaluation (tailored intervention). Modifications were made based on an analysis of the patient’s previous experiences, their current readiness for change, and the availability of time and emotional resources. The process of tailoring materials and tasks was based on the patient’s subjective feelings, the physical therapist’s professional opinion, and the principles of motivational interviewing. The dietitian used techniques such as open-ended questions, affirmation, reflection, and summarization, which allowed the pace of change to be adapted to the patient’s current level of readiness. The entire process was carried out within a model of partnership and shared decision-making, which ensured that the difficulty level of the tasks was tailored to the participants’ individual capabilities and supported the development of autonomous motivation.

To prevent missed appointments, any necessary rescheduling was handled by moving appointments to the earliest possible date that was convenient for the patient, which helped ensure that participants did not fall out of the schedule and maintained full attendance. Patients kept continuous food diaries throughout the entire intervention period, submitting them in their preferred format: on an ongoing basis before meals, in batches every 3 days, or on a weekly basis. Regarding the lack of standardized, aggregated data on physical activity, we would like to clarify that—apart from physical therapy goals—the exercise regimen was determined individually based on the principles of motivational interviewing (e.g., by gradually increasing the daily number of steps or choosing a preferred form of activity), and fitness trackers and mobile apps with GPS and an accelerometer were used for self-monitoring. Due to the process-oriented and highly individualized nature of these goals, as well as their ongoing evaluation and modification during subsequent visits, these data were qualitative in nature. Consequently, quantitative aggregation and averaging of physical activity levels for the entire sample would be methodologically unjustified and were not included in the statistical analysis. If needed, the habit chain was tailored to the individual needs of the patient. It could also include tracking the number of vegetable servings consumed daily, the number of meals containing protein, hours of sleep, stress levels, regular supplementation, pharmacotherapy, body brushing or massage, and other relevant health aspects. The habit chain could be shortened, left in its original format, or expanded with new habits depending on the patient’s needs. Collaboration with the patient was based on Motivational Interviewing (MI), a therapeutic approach focused on supporting patients in making health behavior changes by building internal motivation and engagement in the treatment process [10]. The intervention diet was developed in accordance with the recommendations for healthy adults in Poland described in the “Nutrition Standards for the Polish Population,” edited by M. Jarosz, based on the 2018 edition. The diet was continuously updated in response to subsequent amendments to these standards (2020, 2022) [11].

The caloric value of the diet was individually reduced relative to the participants’ estimated total daily energy expenditure (TDEE), which was determined based on the Resting Energy Expenditure (REE) calculated using the Mifflin–St Jeor formula, adjusted for physical activity level (PAL) according to the guidelines of the Institute of Food and Nutrition in Poland.

Formulas:

- Mifflin Formula (Women): REE (resting energy expenditure kcal) = (10 × body weight [kg]) + (6.25 × height [cm]) − (5 × [age]) − 161
- Mifflin Formula (Men): REE (resting energy expenditure kcal) = (10 × body weight [kg]) + (6.25 × height [cm]) − (5 × [age]) + 5
- Total daily energy expenditure (TDEE) = estimated resting energy expenditure (REE) × PAL

This diet was characterized by a carbohydrate content of 30–45% of the total energy value, with simple sugars limited to below 10%. The protein intake was 25–35%, corresponding to 1.4–1.8 g/kg of body weight. The energy from fats accounted for 25–35% of the total energy value of the diet, with saturated fatty acids not exceeding 5%. The fiber content ranged from 30 to 40 g. The diet was developed using the Aliant program [12], which was based on a detailed food composition database created by the United States Department of Agriculture (USDA) [13]. In the study, the diet break method was implemented in cases of weight stagnation. The suspension period lasted from 2 to 4 weeks and involved maintaining a zero-energy balance and increasing carbohydrate intake. The author’s method was based on five fundamental principles. The first was an individually tailored diet considering, among other things, taste preferences, quantity, and mealtimes. The second aspect was physical activity adjusted to the participants. The third was consistent contact with a dietitian. The fourth was an emphasis on acquiring healthy eating habits. The last was the application of psychodietetic solutions based on proprietary materials. For each patient for whom psychological support seemed beneficial, a consultation with a certified psychotherapist was recommended. Patients requiring specialized care for movement disorders were referred to a qualified physiotherapist or orthopedist to ensure a comprehensive approach to treatment.

### 2.4. Data Collection

The study was conducted from 2018 to 2024. As part of the research, participants were classified according to medical diagnosis, including metabolic disorders such as endometriosis, polycystic ovary syndrome (PCOS), insulin resistance, hypothyroidism, type 2 diabetes, gout, food intolerances, and other gastrointestinal disorders. Additionally, hypertension and cardiovascular diseases were considered, including hypercholesterolemia, hypertriglyceridemia, and chronic circulatory failure. The classification also included chronic kidney failure, gastroesophageal reflux disease, and musculoskeletal system pathologies. Analyses of the impact of comorbidities were pre-specified, whereas additional analyses concerning body-weight fluctuations were exploratory in nature.

The study also identified individual cases of diseases that did not recur among other participants. During the initial visit with a dietitian, a body composition analysis was performed using a bioelectrical impedance analysis (BIA) device. The InBody270 body composition analyzer, produced by InBody (Seoul, Republic of Korea), utilized an 8-point tetrapolar contact electrode system. Technical specifications included frequencies of 20 kHz and 100 kHz, a current intensity of 200 µA, and a maximum body weight limit of 250 kg [14]. Each participant received detailed instructions before beginning the analysis, and compliance with the instructions was verified immediately before the test. Measurements were taken after the bladder had been completely emptied, with participants refraining from consuming fluids immediately before the analysis, after at least 24 h of abstinence from alcohol and no intense physical exertion, and taking into account the phase of the menstrual cycle in women. The analysis measured body weight, total water content, fat mass, and skeletal muscle mass. The parameters also included visceral fat levels. During the bioimpedance body composition measurement, the results of selected parameters were classified into three categories: above the norm (above the upper measurement limit), below the norm (below the lower measurement limit), and within the norm (within the lower and upper measurement limits). Norm ranges, exact formulas, and their estimations were directly determined by the InBody270 body composition analyzer. This device met European standards, and the normative points were generated and individually adjusted to the patient by the device itself. The calculation method is proprietary to the manufacturer, who does not disclose the statistical formulas. Body mass index (BMI) norms were adopted according to the then-current WHO guidelines, which specify a healthy BMI range of 18.5–24.9 for both sexes [15]. Body composition measurements were conducted at regular intervals (every 2–3 weeks, with a maximum two-month gap between measurements). The collected data were then analyzed to monitor changes in patients’ body composition. After the first visit, patients had a scheduled follow-up consultation within a week. During the initial control visit, participants received a dietary plan. The plan and dietary strategy were discussed in detail with each patient, and subsequent follow-up visits occurred approximately every 2–3 weeks, with frequency individually determined. During each follow-up visit, a full set of body composition measurements was performed. The total duration of patients’ participation in the program ranged from 2 to 12 months (with a mean treatment duration of 6 months; SD = 4.04 months), which corresponded to an estimated 8–12 face-to-face consultations per participant.

### 2.5. Study Design

**Figure 1:**
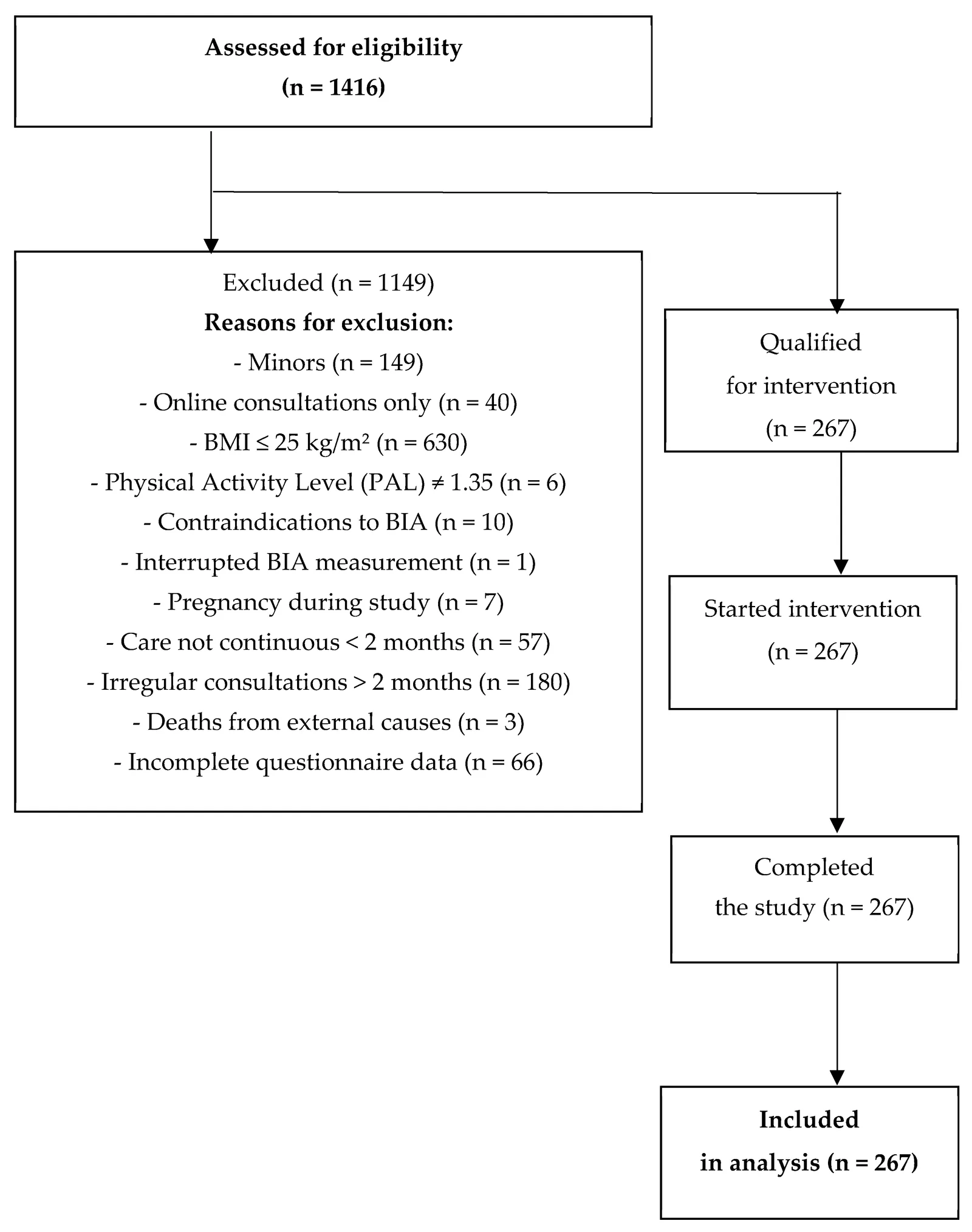
Flowchart demonstrating the selection of study participants.

This study was conducted in accordance with the TREND (Transparent Reporting of Evaluations with Nonrandomized Designs) guidelines. The completed TREND checklist is included as Supplementary Materials, while detailed participant selection data are presented in Figure 1.

### 2.6. Statistical Analysis

The first stage of the analysis of the collected research data was to test the normality of the distribution of the variables using the Kolmogorov–Smirnov test with the Lilliefors correction, both overall and by subgroup. In addition, the homogeneity of variances was verified using Levene’s test. Depending on the results of these preliminary analyses, either parametric or nonparametric methods were selected for further calculations. When the assumptions for analyzing dichotomous quantitative and qualitative variables were met, Student’s *t*-test was used; when they were not met, the Mann–Whitney U test was used. The Kruskal–Wallis test was used to assess the relationship between a quantitative variable and a qualitative variable with more than two categories.

When analyzing correlations between quantitative variables, only Spearman’s rank correlation was used because at least one of the variables did not meet the normality assumption. For the analysis of variables measured on qualitative scales, the independence test was used, with Yates’s correction for continuity applied in situations where the condition for the applicability of this test was not met due to low expected frequencies in the two-way contingency table. For repeated measurements, the Wilcoxon signed-rank test was used, which is the nonparametric equivalent of the *t*-test for paired samples, when the use of a parametric test was not justified due to the lack of normality in the distribution.

The main analysis was evaluated using a predefined basic threshold for statistical significance of *p* < 0.05. To balance the control of the Type I error rate with the minimization of the risk of increasing the Type II error rate (which could lead to overly conservative exclusion of clinically significant results), no formal corrections for multiple comparisons (Bonferroni corrections) were applied in the main results tables. However, to assess the stable dependencies bordering on significance, a post hoc Holm sequential procedure was applied. Although some biological parameters (e.g., body composition indices) slightly exceeded the *p* < 0.05 threshold after applying the Holm correction—which reflects the expected increase in Type II error sensitivity with a rigorous correction—the resulting corrected *p*-values remained in close proximity to the significance threshold, confirming their overall stability in the analyses conducted.

The effect size for nonparametric tests was expressed using the coefficient. In accordance with standard practice for reporting nonparametric statistics, standard confidence intervals are not provided for this measure. The results are presented using descriptive statistics (means, standard deviations, quartiles, interquartile ranges, minimum and maximum values, and frequencies and percentages). Graphical presentations were prepared using MS Excel 2013, and statistical calculations were performed using the IBM SPSS Statistics software package (version 22.0; IBM Corp., Armonk, NY, USA).

## 3. Results

### 3.1. Study Participants

The study group consisted of 267 people. In the first stage of the study, participants were selected from a group of 1416 patients who attended dietary consultations. After a thorough analysis of the initial criteria, minors (*n* = 149) and those who used only online consultations (*n* = 40) were excluded, reducing the pool of potential candidates to 1227. The main inclusion criterion was overweight or obesity, as defined by body mass index. The BMI cutoff points for overweight and obese individuals were 25 kg/m^2^ and 30 kg/m^2^, respectively, regardless of comorbidities. Based on this, 630 people were excluded, leaving 597 eligible participants. The data were then filtered to include only valid electrical bioimpedance analysis measurements obtained under strictly standardized pre-test conditions. This criterion led to the elimination of one participant. The study group was narrowed down to 596 participants. The physical activity level (PAL) was defined as low (1.30–1.69) and set strictly at PAL = 1.35. Six participants who did not meet this exact threshold (PAL < 1.35 or >1.35) were excluded, which further reduced the study group to 590 individuals. In the next stage of selection, 7 women who became pregnant during the study were removed. The study group thus shrank to 583 patients. Subsequently, 10 participants were excluded due to contraindications to BIA for body composition assessment. These factors reduced the number of eligible patients to 573. Additionally, 57 people who did not receive continuous dietary care for the required minimum of 2 months were excluded, reducing the potential candidate pool to 516. Another criterion was maintaining uninterrupted contact with the dietitian and regular visits, with a maximum interval of 2 months between measurements. As a result, 180 people were excluded based on this criterion, reducing the participant pool to 336. During data collection, three deaths from various causes were recorded, which further reduced the number of participants to 333. Participants also completed a questionnaire during the study. Respondents who did not fully complete the questionnaire were excluded from the participant pool (*n* = 66), bringing the final study sample to 267 participants. Participants’ ages ranged from 18 to 74 years, with an average of 40.06 ± 11.52 years. Among the participants, 73.4% (*n* = 196) were women, and 26.6% (*n* = 71) were men, with average ages of 40.36 ± 11.68 years and 39.24 ± 11.09 years, respectively. The largest age group was people aged 30–39 (34.8%, *n* = 93). Due to the retrospective nature of the study, which was based on records from routine clinical practice, complete clinical data and body composition parameters were collected only for patients who completed the initial diagnostic phase and continued with treatment. For individuals excluded at an early stage (due to early withdrawal or loss of contact), baseline data were incomplete or unrecorded, which prevented reliable statistical comparisons and a formal sensitivity analysis for the entire initial sample. A detailed qualification for the study is presented in Figure 1.

The average body weight of the examined individuals before the nutritional and behavioral intervention was 93.81 ± 18.62 kg, ranging from 63 to 190.4 kg. The total amount of water in the body averaged 41.06 ± 8.40 kg, with a range of 35.00–73.30 kg. The results of individual body composition measurements using bioimpedance were as follows: fat tissue mass (BFM) 37.80 ± 12.26 kg, lean body mass (FFM) 55.98 ± 11.40 kg, skeletal muscle mass (31.34 ± 6.92 kg), body mass index 32.72 ± 5.59 kg/m^2^, body fat percentage (PBF) 39.90 ± 7.66%, target body weight 70.57 ± 11.41 kg, visceral fat level (VFL) 16.91 ± 4.84 points, and skeletal muscle mass index (SMI) 8.30 ± 1.16 kg/m^2^. All patients had confirmed body weight and BMI values above the normal range, and nearly all individuals had BFM (98.9%, *n* = 264) and PBF (98.9%, *n* = 264) indicators above the normal range. More than half of the participants in the strategy achieved above-normal results for skeletal muscle mass (SMM) (61.0%, *n* = 163), FFM (59.6%, *n* = 159), or TBW (58.1%, *n* = 155). At baseline, 35.2% of participants had overweight, with first-degree obesity in 37.1% (*n* = 99), second-degree obesity in 17.6% (*n* = 47), and third-degree obesity in 10.1% (*n* = 27). The most common conditions among the participants were insulin resistance (24.0%, *n* = 64), hypertension (21.7%, *n* = 58), and hypothyroidism (18.0%, *n* = 48). An analysis of the multimorbidity index showed that the number of diagnosed diseases ranged from 0 to 7, with an average of 1.26 ± 1.25 diseases per person. The most frequently observed were one (30.7%, *n* = 82) or two (23.2%, *n* = 62) conditions.

The study found that the percentage of daily requirements was 80%, meaning the calorie reduction in the diet was 20% (ranging from 12.2% to 28.4%). Total energy intake varied significantly depending on the gender of the participants. In the group of men, it ranged from 1600 to 3000 kcal (mean: 2107.0 ± 216.7 kcal; median: 2100 kcal), while in the group of women, it ranged from 1400 to 2300 kcal (mean: 1690.3 ± 163.5 kcal; median: 1700 kcal). The “diet break” strategy was used in 7.9% (*n* = 21) of patients. In each case, applying this strategy resulted in further weight loss. Individuals who used the “diet break” method lost weight at a rate similar to those on traditional reduction. The collaboration with a dietitian lasted an average of 6.24 ± 4.04 months, with the most common period being from 4 to 6 months (36.7%, *n* = 98), with the shortest collaboration lasting 2 months and the longest lasting a year.

### 3.2. Assessment of the Effectiveness of Behavioral–Nutritional Intervention in Weight Reduction and Its Composition

The results of body composition measurements obtained by bioelectrical impedance analysis before and after nutritional and behavioral intervention were analyzed. Initial body weight values ranged from 63 kg to 190.4 kg. The average weight loss was 10.45 ± 7.56 kg. Most patients managed to reduce their body weight by 1–10% (52.1%, *n* = 139). Additionally, 38.6% of participants (*n* = 103) achieved a 10–20% reduction, 6.7% (*n* = 18) a 20–30% reduction, and 1.5% (*n* = 4) over a 30% reduction. In 1.1% (*n* = 3) of participants, no weight reduction was observed, and their weight increased. Overall, 88% of patients achieved a weight reduction of 5% relative to their initial value. The average weight loss in kilograms was as follows: up to 10%—6.33 kg, 10 to 20%—12.61 kg, 20 to 30%—24.06 kg, and for the four individuals who reduced their weight by more than 30%, the average loss was 45.23 kg. For the three individuals who gained weight, the increase was an average of 0.63 kg. Significant changes in body composition were also observed: total body water decreased by 1.30 ± 1.65 kg, fat tissue mass by 8.70 ± 6.76 kg, and fat-free mass by 1.72 ± 2.20 kg. Losses in SMM averaged 1.11 ± 1.36 kg, and BMI decreased by 3.62 ± 2.46 kg/m^2^. Detailed results of the analysis are presented in Table 1.

**Figure 2:**
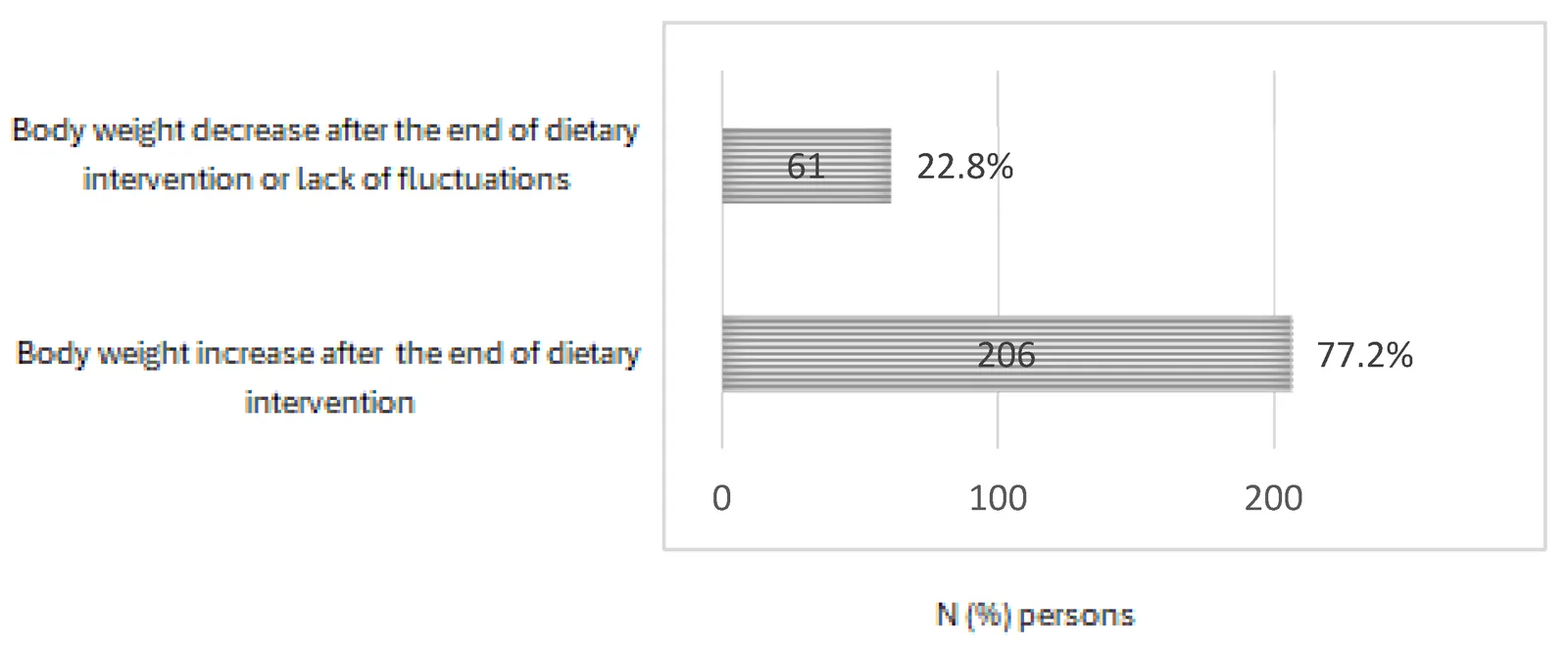
Body weight decrease after the end of dietary intervention or lack of fluctuations.

The effects of the strategy were also assessed as the percentage change of selected parameters relative to the baseline value before the intervention. The highest percentage effect of the intervention was observed in the VFL index (24.46 ± 17.62%) and BFM (23.04 ± 13.84%). Patients managed to reduce PBF levels by 14.31 ± 11.39%, MC by 10.79 ± 6.12%, and BMI by 10.78 ± 6.13%. The remaining evaluated parameters decreased by less than 10% compared to the baseline values. The qualitative changes in selected parameters after the intervention were also assessed, verifying in which patient groups a positive effect was achieved (parameter decrease), in which group parameters did not change after the nutritional intervention, and in how many patients there was an increase in selected parameters despite the intervention. The largest qualitative effects of the intervention concerned BMI (99.3%, *n* = 265), MC (98.9%, *n* = 264), BFM (98.5%, *n* = 263), PBF (96.6%, *n* = 258), and VFL (92.9%, *n* = 248). Negative correlations were also demonstrated between energy deficit relative to total daily energy requirements and the quantitative change in parameters such as MC (*p* < 0.0001), TBW (*p* = 0.0499), BFM (*p* = 0.0006), SMM (*p* = 0.0343), and BMI (*p* = 0.0082). Statistically significant correlations between energy deficit and the percentage effect of the strategy were not confirmed. When measuring body weight after the active reduction phase, an average weight gain of 5.38 ± 8.62 kg was confirmed, which applied to most patients, as illustrated in Figure 2.

### 3.3. The Impact of Metabolic Disorders on the Effects of the Strategy

Patients diagnosed with insulin resistance achieved greater quantitative effects of the strategy in terms of TBW (*p* = 0.0322), FFM (*p* = 0.0343), SMM (*p* = 0.0210) and SMI (*p* = 0.0039). Patients with insulin resistance showed a higher percentage effect after the intervention in terms of TBW, FFM and SMM, while patients without insulin resistance achieved a higher effect in terms of PBF, as shown in Table 2.

Patients diagnosed with gout achieved better quantitative effects of dietary therapy in relation to TBW (*p* = 0.0200), FFM (*p* = 0.0252), and SMM (*p* = 0.0493). Additionally, patients with gout achieved a higher percentage of the nutritional intervention effect concerning TBW (*p* = 0.0266) and FFM (*p* = 0.0325).

Patients with hypertension achieved higher effects from the strategy in parameters such as TBW (*p* = 0.0005), FFM (*p* = 0.0014), and SMM (*p* = 0.0017). Participants without hypertension achieved higher effects in the PBF parameter (*p* = 0.0081). Analyzing the percentage effect of the nutritional and behavioral intervention, it was confirmed that patients with hypertension achieved higher effects in TBW, FFM, and SMM. Conversely, individuals without hypertension achieved higher percentage effects in BFM, PBF, and VFL. The percentage effect of the intervention is shown in Table 3.

Considering comorbidities associated with excess body weight, no statistically significant differences were confirmed between the quantitative and percentage effects of interventions and the incidence of type 2 diabetes, PCOS, endometriosis, and hypothyroidism. The effects of the interventions did not depend on whether patients had been diagnosed with cardiovascular diseases, gastroesophageal reflux, or food intolerances. There were no statistically significant differences between the effectiveness of the interventions in patients who have excessive weight but have no comorbidities and those with at least one diagnosed condition. Analyzing the percentage effects of the interventions, it was confirmed that healthy individuals achieved higher effects in parameters such as BFM (*p* = 0.0118), PBF (*p* = 0.0009), and VFL (*p* = 0.0053). It was confirmed that patients without other diseases achieved higher percentage effects of the intervention in BFM (*p* = 0.0219), PBF (*p* = 0.0139), and VFL (*p* = 0.0218). People with a higher baseline BMI before starting the intervention achieved higher quantitative effects after the nutritional and behavioral intervention in parameters such as MC (*p* = 0.0001), TBW (*p* = 0.0011), BFM (*p* < 0.0001), FFM (*p* = 0.0023), SMM (*p* = 0.0009), BMI (*p* < 0.0001), and SMI (*p* < 0.0001). Analyzing the percentage effect of the strategy, it was shown that individuals with a higher BMI before the nutritional intervention achieved higher effects in parameters such as MC (*p* = 0.0083), TBW (*p* = 0.0062), FFM (*p* = 0.0131), SMM (*p* = 0.0069), BMI (*p* = 0.0146), and SMI (*p* = 0.0001).

### 3.4. Assessment of Long-Term Body Weight Stability

Considering the adopted criterion of no fluctuation, i.e., maintaining a weight loss of at least 10% for a minimum of one year after completing therapy, 23.2% of the participants achieved this goal. It was confirmed that the change in BMI resulting from dietary intervention showed a significant correlation with weight fluctuation. Individuals who lost more kilograms (R = 0.361; *p* < 0.0001) or a higher percentage of BMI (R = 0.385; *p* < 0.0001) were more susceptible to greater weight regain. Weight fluctuation affected those who achieved a higher BMI change, both in kilograms (*p* = 0.0004) and percentage (*p* < 0.0001).

## 4. Discussion

Obesity-related mortality is rising globally, and identifying dietary–behavioral strategies that produce durable weight loss remains an urgent clinical priority. This study evaluated the nutritional status of adults with excess body weight following a dietetic–behavioral intervention. The intervention met its primary hypothesis: participants achieved statistically significant reductions in body weight and meaningful improvements in body composition. The average weight loss was 10.45 ± 7.56 kg. In the ≤10% loss group, the average was 6.33 kg; in the 10–20% group, 12.61 kg; in the 20–30% group, 24.06 kg; and among those exceeding 30% loss, 45.23 kg. The clinically significant therapeutic outcome—defined as a reduction of at least 5% of initial body weight—was achieved by 88% of participants.

Compositional outcomes were equally encouraging. Fat tissue mass decreased by 8.70 ± 6.76 kg, while the average reduction in lean mass was 1.72 kg (±2.20 kg), and skeletal muscle mass decreased by 1.11 ± 1.36 kg. Although fat loss accounted for the majority of total weight loss, this process was also accompanied by a moderate but statistically significant loss of lean body mass.

Rapid weight loss, particularly of lean tissue, can trigger metabolic adaptations that impede further reduction and long-term maintenance [16], so preserving lean mass is a key metric of intervention quality. Current guidelines from the Polish Obesity Treatment Society (PTLO) state that a weight loss of at least 5% constitutes a threshold of clinical significance, inducing measurable health benefits. Although pharmacotherapy and newly developed bariatric procedures have been shown to be effective in reducing body weight and regulating metabolic parameters, behavioral and dietary interventions remain the fundamental and safest first-line treatment option, free from the risk of surgical complications or adverse drug reactions [17]. Furthermore, the results of our own studies partially confirm the hypothesis that a patient’s clinical profile influences the effectiveness of the intervention. Among participants with insulin resistance, the intervention produced significantly greater reductions in fat-free mass (*p* = 0.0343) and skeletal muscle mass (*p* = 0.0210). Similar results were shown in the study by Bartholomew et al., which involved a cohort of 57 obese patients subjected to an 8-week weight stabilization, followed by an 8-week intervention with an energy deficit. Of the mean 6.8 ± 3.2 kg lost, fat-free mass accounted for 1.5 ± 2.6 kg; notably, greater baseline insulin sensitivity independently predicted smaller FFM losses [18]. Among the present cohort, the absence of insulin resistance was associated with greater reductions in percentage body fat (*p* = 0.0270) and visceral fat (*p* = 0.0261). Insulin resistance is not classified as a separate diagnosis in the International Classification of Diseases (ICD-10); rather, it is recognized as a condition predisposing to other metabolic disorders, and its diagnosis remains clinically imprecise, with no standardized definition in current guidelines [19]. Endocrine disorders can impair lipolysis and impede fat loss through disrupted biochemical and hormonal regulation [20,21]. Hypothyroidism, resulting from thyroid hypofunction, decreases thermogenesis, inhibits triglyceride hydrolysis, and increases water and electrolyte retention—each contributing independently to weight gain [22]. Samuels et al. [23] reported that each 10 pg/dL rise in free triiodothyronine (FT3) corresponded to a 0.15 kcal/kg/day increase in resting metabolic rate (RMR), equivalent to 10.5 kcal/day in a 70 kg individual. Although hypothyroidism is widely considered to attenuate weight loss, the present study found no statistically significant differences in absolute or relative intervention outcomes between participants with and without the condition. Similar patterns have been reported indicating a correlation between the degree of body fat reduction and polycystic ovary syndrome. A study of 57 women—37 with PCOS and 20 without—examined differences in body composition and metabolic parameters despite comparable baseline anthropometric indicators (age, BMI, body weight) [24]. DXA analysis revealed that women with PCOS had significantly greater baseline trunk fat mass than the control group; the PCOS group also showed significantly higher values for fasting glucose, fasting insulin, HOMA-IR, and glycated hemoglobin (HbA1c). Despite these baseline differences, the present study found no statistically significant differences in absolute or relative outcomes of the nutritional–behavioral intervention between participants with and without PCOS.

The present study found a differential response to the intervention based on hypertension status. Participants without hypertension achieved greater reductions in percent body fat, while hypertensive participants showed greater preservation or increases in total body water, lean body mass and skeletal muscle mass. These differences may reflect hypertension-related mechanisms, including upregulation of the renin–angiotensin–aldosterone system (RAAS), endothelial dysfunction, increased vascular stiffness, and chronic inflammation [25,26].

Effective obesity management demands both successful initial weight reduction and sustained long-term weight stability. A well-documented phenomenon in scientific literature, the “yo-yo effect” refers to recurring cycles of weight loss and regain that frequently follow the discontinuation of dietary interventions In the absence of a consensus definition establishing a threshold time frame for regain, any post-loss weight gain is broadly considered to qualify as the yo-yo effect [27]. Wing and Hill [28] define successful weight loss maintenance as a voluntary reduction of at least 10% of initial body weight sustained for a minimum of one year. By this criterion, only 23.2% of participants in the present study achieved long-term success—indicating that the majority did not maintain their results. This finding is consistent with broader literature reporting that weight regain affects 70–90% of individuals who lose more than 10% of body weight intentionally [29]. Among individuals who intentionally lost more than 5% of body weight, approximately one-third experienced the yo-yo effect. Weight regain typically begins within the first year, with pre-intervention weight restored or exceeded within 2–5 years [30,31]. Weight stability during that initial year is therefore a key predictor of long-term success. Wing and Hill further reported that sustaining weight loss for at least two years halves the risk of full weight regain [28].

In the present study, the magnitude of BMI reduction was significantly correlated with subsequent yo-yo susceptibility: participants who lost more kilograms (R = 0.361; *p* < 0.0001) or achieved a greater percentage reduction in BMI (R = 0.385; *p* < 0.0001) were more prone to regain. A marginally significant association (*p* = 0.0256) was also observed between post-intervention BMI and yo-yo occurrence, with higher post-intervention BMI values associated with greater susceptibility. A 2024 study published in Nature by Hinte et al. [32] provided a molecular basis for this susceptibility, demonstrating that adipose tissue retains epigenetic and transcriptional changes induced by obesity even after substantial weight loss—effectively priming adipocytes for accelerated regain upon re-exposure to an obesogenic environment. Hinte et al. propose that weight fluctuation in individuals with a history of obesity reflects persistent transcriptional and epigenetic reprogramming of adipocytes. Notably, adipocytes were found to retain epigenetic memory of their prior hypertrophic state both before and up to two years following bariatric surgery—underscoring the durability of these cellular changes. This adipocyte-level reprogramming may promote ectopic lipid deposition in skeletal muscle tissue, driving the development of insulin resistance and lipotoxicity—processes that represent a key pathophysiological basis for the difficulties observed in maintaining stable body weight and the characteristic tendency toward cyclical fluctuation [33]. Cyclical body weight fluctuations involving repeated cycles of loss and regain are associated with adverse changes in body composition, including a progressive increase in fat tissue mass accompanied by a corresponding decrease in lean mass. Kroeger et al. [34] confirmed that even a modest weight regain of approximately 3–6% following an initial reduction of 10% is sufficient to negate the health benefits previously achieved through weight loss. Additionally, this pattern of weight cycling substantially increases the risk of sarcopenic obesity, a condition characterized by the simultaneous loss of muscle mass and accumulation of fat tissue [35]. Yates et al. [36] reported that individuals who lost weight and subsequently regained it lost a mean of 5.23 kg of fat mass during the first year of follow-up, with the full amount recovered between months 12 and 24. Concurrently, a mean loss of 1.88 kg of lean body mass was observed over the same period, yielding a net lean mass deficit of approximately 1 kg across the two-year observation window. Evidence also suggests that the yo-yo effect may correlate with heightened systemic inflammation, with emerging data indicating possible links between cyclical weight oscillations and an elevated risk of metabolic disorders including type 2 diabetes, cardiovascular disease, and hypertension [37]. These observations suggest that weight cycling has a potentially stronger association with all-cause mortality than does persistent obesity. The underlying mechanism remains poorly understood, though immune dysregulation has been proposed as a contributing factor. Weight fluctuations have also been associated with greater severity of depressive symptoms and worsening cardiometabolic parameters, including blood pressure, lipid profile, insulin, and glucose levels [38,39,40]. Recent evidence further indicates that adjunctive pharmacotherapy with GLP-1 and GIP receptor agonists improves the durability of therapeutic outcomes, with effects sustained for at least four years [41]. However, weight regain frequently occurs following discontinuation of GLP-1 receptor agonists, with approximately two-thirds of lost weight typically recovered within one year [42]—a pattern observed even when conventional dietary counseling is provided concurrently [43].

These findings are consistent with a meta-analysis by Dombrowski et al. [44] encompassing 45 studies and 7788 participants, in which behavioral interventions combining dietary modification with physical activity maintained a mean weight difference of only −1.56 kg (95% CI: −2.27 to −0.86 kg) at 12 months relative to controls. The difficulty of sustaining long-term weight loss is further underscored by evidence indicating that only 25% of patients maintain their achieved weight reduction over time [45]. Flore et al. reported no statistically significant improvement with intensive maintenance-phase interventions (*p* = 0.098). The present study corroborates these challenges, identifying a strong correlation between the rate of BMI reduction and the risk of obesity relapse (*p* < 0.0001), with patients achieving the greatest initial weight loss, averaging 45.23 kg in the >30% reduction subgroup, demonstrating the highest susceptibility to subsequent weight cycling. The evolving understanding of obesity etiopathogenesis reflects a broader reassessment of therapeutic goals, with growing recognition that successful treatment extends beyond initial weight reduction to encompass long-term weight maintenance [46]. The present study’s findings, corroborated by the wider literature, underscore the importance of post-intervention strategies designed to sustain body composition outcomes and mitigate the long-term metabolic consequences of weight regain. These results emphasize the need for continued research aimed at attenuating the deterioration of body composition parameters and reducing susceptibility to weight fluctuation over time.

## 5. Limitations

Despite efforts to maximize the size of the study group, the relatively small number of participants constitutes a significant limitation of this study. Due to the retrospective nature of the analysis, no preliminary sample size calculation was performed, and the study included a purposive sample of patients who completed the full program. The exclusion of individuals who did not comply with the recommendations—resulting from a lack of treatment continuity, unilateral withdrawal, lack of contact with the dietitian, and failure to attend follow-up visits—introduces a risk of selection bias, which limits the ability to directly generalize the results to the entire population of patients visiting the clinic. For logistical reasons and due to the large number of participants, it was not possible to perform all bioelectrical impedance analysis measurements at a fixed time of day or strictly in a fasted state, even though patients were instructed on the importance of such preparation. This issue, along with the impact of changes in total body water on estimates of fat-free mass and skeletal muscle mass, as well as limitations resulting from the use of proprietary algorithms provided by the BIA device manufacturer, may have affected the precision of the obtained body composition readings. Furthermore, the absence of a control group that did not undergo a dietary intervention further limits the ability to unequivocally attribute the observed changes solely to the method used. Concurrent pharmacotherapy is also a confounding factor; the variety of medications taken and modifications to treatment regimens during the study were not systematically accounted for in the analysis, which could have independently influenced metabolic parameters. Furthermore, the lack of standardized longitudinal measurements of cardiometabolic markers (such as lipid profile, blood glucose, or blood pressure) precludes an assessment of direct cardiovascular benefits. Future studies should consider integrating individualized dietary and behavioral interventions with pharmacotherapy and bariatric surgery, with particular attention to the patient’s clinical profile, comorbidities, and baseline health status. Neither hypothyroidism nor PCOS significantly affected intervention outcomes, suggesting broad applicability of the dietary–behavioral approach across patients with these comorbidities. The intervention also effectively preserved lean mass during weight loss. Despite strict adherence to dietary care standards, the high prevalence of weight cycling in the cohort underscores the need for innovative therapeutic protocols targeting the long-term prevention of obesity relapses.

## 6. Conclusions

The use of an integrated dietary and behavioral strategy in patients who had completed the full treatment program was associated with a significant reduction in body weight and BMI, as well as improvements in body composition parameters, indicating the potential utility of this approach in routine outpatient practice. The main component of weight loss was a reduction in body fat, accompanied by a moderate, though statistically significant, loss of lean body mass. The differing dynamics of body composition changes in participants with insulin resistance and hypertension highlight the need to strengthen nutritional education and tailor motivational support to these clinical subgroups. Conversely, the lack of a significant effect of hypothyroidism and polycystic ovary syndrome on the achieved weight loss suggests the possibility of broad application of the described intervention in patients with these comorbidities. Despite adherence to structured standards of dietary care, the high rate of weight regain during the one-year follow-up period (less than a quarter of the participants maintained their weight loss) underscores the complexity of the problem and the need for further research into innovative protocols aimed at the long-term prevention of obesity relapse.

## Figures and Tables

**Table 1:** Quantitative Effect of Nutritional and Behavioral Interventions.

M	SD	Min	Max	Q1	Q2	Q3	*p* (Wilcoxon)	
MC	10.45	7.56	−1.30	66.40	6.10	8.80	12.60	Z = −14.153; *p* < 0.001
TBW	1.30	1.65	−3.30	9.10	0.20	1.10	2.10	Z = −11.169; *p* < 0.001
BFM	8.70	6.76	−0.90	67.20	4.80	7.50	11.10	Z = −14.144; *p* < 0.001
FFM	1.72	2.20	−4.80	12.20	0.30	1.50	2.0	Z = −11.121; *p* < 0.001
SMM	1.11	1.36	−2.40	7.90	0.20	0.90	1.80	Z = −11.559; *p* < 0.001
BMI	3.62	2.46	−0.50	17.80	2.20	3.10	4.30	Z = −14.155; *p* < 0.001
PBF	5.48	4.19	−1.90	28.70	2.70	4.90	7.20	Z = −14.029; *p* < 0.001
VFL	3.98	3.01	−9.00	21.00	2.00	4.00	5.00	Z = −13.598; *p* < 0.001
SMI	0.39	0.45	−2.80	2.90	0.20	0.30	0.50	Z = −12.766; *p* < 0.001

MC—body mass (kg); TBW—total body water (kg); BFM—fat tissue mass (kg); FFM—fat-free mass (kg); SMM—skeletal muscle mass (kg); BMI—body mass index (kg/m^2^); PBF—percentage of fat tissue; VFL—visceral fat level (points); SMI—skeletal muscle mass index (kg/m^2^); M—arithmetic mean; SD—standard deviation; Min—minimum; Max—maximum; Q—quartile; Z—Wilcoxon signed-rank test; *p*—level of statistical significance (*p* < 0.05).

**Table 2:** Quantitative Effect of Long-Term Nutritional and Behavioral Intervention on the Prevalence of Insulin Resistance.

		Insulin Resistance							*p*
		M	SD	Min	Max	Q1	Q2	Q3	
MC	No	10.21	7.35	−1.30	66.40	5.90	8.70	12.50	Z = −0.912; *p* = 0.3620
	Yes	11.22	8.20	0.70	47.50	6.65	9.70	12.70	
TBW	No	1.18	1.63	−3.30	7.20	0.10	0.90	2.10	Z = −2.142; *p* = 0.0322
	Yes	1.66	1.65	−0.40	9.10	0.70	1.40	2.15	
BFM	No	8.60	6.82	−0.90	67.20	4.70	7.60	11.20	Z = −0.434; *p* = 0.6640
	Yes	9.00	6.59	0.50	38.70	5.40	7.40	10.75	
FFM	No	1.56	2.17	−4.80	10.10	0.20	1.30	2.90	Z = −2.116; *p* = 0.0343
	Yes	2.22	2.23	−0.50	12.20	0.85	1.75	2.95	
SMM	No	1.01	1.33	−2.40	6.60	0.10	0.80	1.80	Z = −2.309; *p* = 0.0210
	Yes	1.43	1.40	−0.20	7.90	0.55	1.20	1.80	
BMI	No	3.48	2.28	−0.50	17.80	2.10	3.00	4.20	Z = −1.460; *p* = 0.1442
	Yes	4.05	2.92	0.30	16.40	2.55	3.35	4.40	
PBF	No	5.68	4.39	−1.90	28.70	2.70	5.10	7.40	Z = −1.190; *p* = 0.2340
	Yes	4.86	3.44	−0.10	14.90	2.50	4.55	6.55	
SMI	No	0.35	0.44	−2.80	1.80	0.10	0.30	0.50	Z = −2.890; *p* = 0.0039
	Yes	0.51	0.48	−0.10	2.90	0.30	0.40	0.60	

MC—body mass (kg); TBW—total body water (kg); BFM—body fat mass (kg); FFM—fat-free mass (kg); SMM—skeletal muscle mass (kg); BMI—body mass index (kg/m^2^); PBF—percentage of body fat; SMI—skeletal muscle mass index (kg/m^2^); M—arithmetic mean; SD—standard deviation; Min—minimum; Max—maximum; Q—quartile; Z—Mann–Whitney test; *p*—level of statistical significance.

**Table 3:** Percentage effect of dietary and behavioral intervention on the occurrence of hypertension.

		Hypertension							*p*
		M	SD	Min	Max	Q1	Q2	Q3	
MC	No	10.85	6.01	−1.40	35.34	7.04	9.88	13.16	Z = −0.709; *p* = 0.4782
	Yes	10.58	6.58	−0.40	34.87	6.71	8.73	14.27	
TBW	No	2.65	3.47	−7.32	14.60	0.32	2.45	4.57	Z = −3.271; *p* = 0.001
	Yes	4.23	3.61	−6.35	12.28	1.55	4.75	6.53	
BFM	No	23.91	13.52	−1.46	67.55	15.16	22.83	30.63	Z = −2.362; *p* = 0.018
	Yes	19.89	14.62	−2.33	74.09	10.89	15.88	27.77	
FFM	No	2.63	3.45	−7.49	14.62	0.42	2.46	4.57	Z = −2.935; *p* = 0.003
	Yes	3.96	3.55	−6.78	12.23	1.64	4.41	6.16	
SMM	No	3.05	3.76	−7.72	16.71	0.44	2.64	5.18	Z = −2.958; *p* = 0.003
	Yes	4.50	3.79	−5.99	13.47	1.84	5.35	6.77	
BMI	No	10.83	6.01	−1.39	35.27	7.04	9.77	13.18	Z = −0.664; *p* = 0.507
	Yes	10.58	6.58	−0.71	34.83	6.79	8.77	14.00	
PBF	No	15.19	11.19	−1.62	55.76	7.62	13.20	20.32	Z = −2.919; *p* = 0.004
	Yes	11.14	11.64	−5.76	60.17	3.27	9.01	15.87	
SMI	No	4.36	5.32	−43.08	20.71	2.06	3.85	6.67	Z = −0.760; *p* = 0.447
	Yes	4.81	4.16	−7.78	17.80	1.37	4.85	6.86	

MC—body mass (kg); TBW—total body water (kg); BFM—fat tissue mass (kg); FFM—fat-free mass (kg); SMM—skeletal muscle mass (kg); BMI—body mass index (kg/m^2^); PBF—percentage of fat tissue; SMI—skeletal muscle mass index (kg/m^2^); M—arithmetic mean; SD—standard deviation; Min—minimum; Max—maximum; Q—quartile; Z—Mann–Whitney test; *p*—level of statistical significance (*p* < 0.05).

## Data Availability

The data presented in this study are available from the corresponding author upon reasonable request. The data are not publicly available due to privacy and ethical restrictions.

## Supplementary Materials

The following supporting information can be downloaded at: https://www.mdpi.com/article/10.3390/nu18172814/s1, TREND Statement Checklist. Ref. [47] has been cited in Supplementary Materials.
